# Ocean-Surface Wave Measurements Using Scintillation Theories on Seaborne Software-Defined GPS and SBAS Reflectometry Observations

**DOI:** 10.3390/s23136185

**Published:** 2023-07-06

**Authors:** Lung-Chih Tsai, Hwa Chien, Shin-Yi Su, Chao-Han Liu, Harald Schuh, Mohamad Mahdi Alizadeh, Jens Wickert

**Affiliations:** 1 Center for Space and Remote Sensing Research, National Central University, Taoyuan 320317, Taiwan; 2 Department of Space Science and Engineering, National Central University, Taoyuan 320317, Taiwan; 3 Graduate Institute of Hydrological and Oceanic Sciences, National Central University, Taoyuan 320317, Taiwan; 4 Aacdemia Sinica, Taipei 115024, Taiwan; 5 Institute of Geodesy and Geoinformation Science, Technische Universität Berlin, 10553 Berlin, Germanywickert@gfz-potsdam.de (J.W.); 6 Helmholtz Centre Potsdam, German Research Centre for Geosciences GFZ, 14473 Potsdam, Germany; 7 Department of Geodesy and Geomatics Engineering, K. N. Toosi University of Technology, Tehran 19697, Iran

**Keywords:** GPS/GNSS reflectometry, ocean-surface wave measurement, radio scintillation, software-defined GPS receiver

## Abstract

In this study, a low-cost, software-defined Global Positioning System (GPS) and Satellite-Based Augmentation System (SBAS) Reflectometry (GPS&SBAS-R) system has been built and proposed to measure ocean-surface wave parameters on board the research vessel New Ocean Researcher 1 (R/V NOR-1) of Taiwan. A power-law, ocean-wave spectrum model has been used and applied with the Small Perturbation Method approach to solve the electromagnetic wave scattering problem from rough ocean surface, and compared with experimental seaborne GPS&SBAS-R observations. Meanwhile, the intensity scintillations of high-sampling GPS&SBAS-R signal acquisition data are thought to be caused by the moving of rough surfaces of the targeted ocean. We found that each derived scintillation power spectrum is a Fresnel-filtering result on ocean-surface elevation fluctuations and depends on the First Fresnel Zone (FFZ) distance and the ocean-surface wave velocity. The determined ocean-surface wave speeds have been compared and validated against nearby buoy measurements.

## 1. Introduction

The Global Positioning System (GPS) or Global Navigation Satellite Systems (GNSS), which was first conceived for the purpose of navigation, has been successfully used as an Earth remote sensing tool due to the multipath effects of being reflected or scattered over the Earth’s surface. Such technique is commonly referred to as GPS reflectometry (GPS-R) or GNSS reflectometry (GNSS-R). Some typical approaches of the GPS/GNSS-R technique have been proposed for remote sensing applications to ocean altimetry and sea state measurement [1,2,3,4,5,6]. Basic understanding of GPS/GNSS-R signal properties from an air- or space-borne receiver over the ocean is based on fairly extensive theories developed by Zavorotny and Voronovich [3]. When the GPS signal impinges on an irregular surface, e.g., rough sea or ocean surface, it is not only reflected in a specular direction but also scattered in other directions from the surface. One specific area on the ocean surface forming a signal footprint stretching between the transmitter and receiver, which contributes to the overall received signal power. For an air- or space-borne GPS/GNSS-R system, the receiving signal footprint range is about the order of receiver altitude and could approach a few tens or hundreds of kilometers, respectively. The observation products include time-delay waveforms with trailing edge or characterized delay-Doppler maps (DDM) as a two-dimensional power distribution function: one coordinate is the GPS signal propagation time delay, and the other coordinate is the Doppler shift, with respect to the GPS/GNSS carrier frequency. The obtained structure of the trailing time-delay waveform or horseshoe-shaped contour power distribution from DDM can be used to retrieve the sea/ocean surface wave and current velocities.

In this study, a seaborne GPS&SBAS-R system is proposed to be used for ocean-surface state measurements. However, the corresponding observations have much smaller signal footprints over the ocean because of a low receiver altitude and cannot provide the typical time-delay waveform or DDM data product. We propose to complement these developed electromagnetic wave-scattering theories on solving the seaborne GPS/GNSS-R problem from a different perspective by radio scintillation theory. When a radio wave is transmitted from a GPS or GNSS satellite onto a sea or ocean, its wave front will be distorted by a moving rough surface. Such rough surface consists of irregularly spatial and temporal variations, as sea/ocean waves generally exist over a range of scales, from swell to gravity and even capillary waves [7]. If the irregularity scale sizes are comparable or smaller than the first Fresnel zone (FFZ), then scattering effects can be seen. In such a condition, after the radio wave has been scattered and emerged from the signal footprint area, its intensity and phase front are randomly modulated and present spatial and temporal fluctuations, what is known as radio scintillation. In this study, we focus on the diagnostics of radio intensity scintillation in the specular direction during seaborne GPS/GNSS-R observations. We recognize these signals as “coherent” scattering signals. In order to understand how ocean surface waves interact with electromagnetic waves and compare to experimental measurements, it is essential to better understand ocean-surface roughness and corresponding spectral-wave models. Furthermore, the scattering of electromagnetic waves from a rough surface can be studied by two general approaches: the Kirchhoff approximation [3,8], and the small perturbation method (SPM) [9,10]. In this study, a power-law, ocean-wave spectrum model has been used and applied with the SPM simulation to compare with experimental GPS/GNSS-R observations.

GPS/GNSS-R measurements usually record the time series of electromagnetic ocean-surface reflection and/or scattering signals as raw data, which can be processed to provide a temporal frequency spectrum of the received signals. In order to measure the signals with respect to short-scale ocean surface waves, e.g., gravity waves, we implemented and developed reflectometry software-defined receivers to receive L1-band Coarse Acquisition (C/A) code signals transmitted from both of GPS and Satellite-Based Augmentation Systems (SBAS) satellites. Compared with usual commercial GPS receivers, software-defined receivers offer added flexibility and versatility by implementing most functions in software [11,12]. Meanwhile, a software-defined GPS receiver can approach a maximum signal acquisition rate of 1000 Hz due to the L1-band C/A code duration at 1 millisecond, and the rate is much higher than that of a typical commercial GPS receiver. 

This paper commences in the next section by introducing the theories as developed for radio scintillations and adapting this to the case of seaborne GPS/GNSS-R observations. Section 3 describes the GPS&SBAS-R system on board the R/V NOR1 and the corresponding signal acquisition and processing. In the subsequent section, we propose the Fourier spectral analysis to derive a breaking frequency and spectral index of obtaining signal intensity spectrum from ocean-surface wave observations. The section also presents the analytical methodology of GPS&SBAS-R measurements on ocean-surface wave speed. The derived ocean-surface wave speeds were compared and validated against in situ buoy measurements. To conclude, we summarize the main conclusions of this paper and point out several problems to be addressed in future studies.

## 2. Basic Theories of Electromagnetic Wave Scattering and Intensity Scintillation Applied on Seaborne GPS/GNSS-R Observations

**Figure 1:**
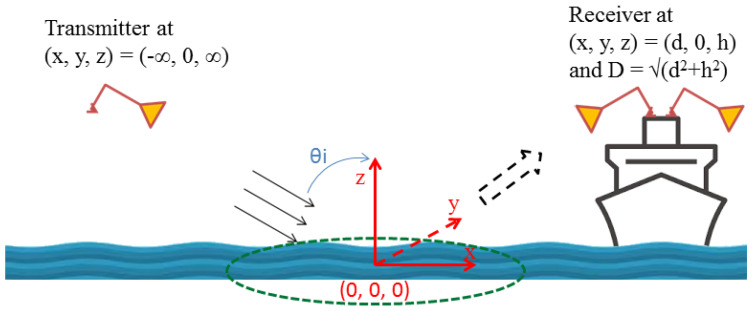
Geometry of a GPS&SBAS-R observation on rough ocean surface. The dash green ellipse represents the corresponding FFZ area on the x-y plane, and the origin is located at the SPP, i.e., the center of FFZ. The GPS&SBAS-R receiver is located at an elevation height of *h* and a horizontal distance of *d* from the SPP; therefore, the total distance *D* is equal to *sqrt*(*d*^2^ + *h*^2^).

In simulating the electromagnetic problem of GPS/GNSS-R signal scattering, we presume that a plane wave *E_i_* incident on a rough ocean surface with an incident angle of *θ_i_* is located on the x-z plane, as shown as the geometry of Figure 1. We note that the footprint of coherent radio-wave reflection and/or scattering over an ocean surface is in practice, estimated by the first Fresnel zone (FFZ) [13,14] as shown as the indicated ellipse on the x-y plane of Figure 1. The FFZ ellipse has a small ratio of *cos*(*θ_i_*) between its semi-minor and semi-major axes; therefore, each seaborne GPS&SBAS-R observation in this study has been treated as a one-dimensional problem along the specular point positions (SPP) over the targeted ocean surface. We define an ocean-surface elevation function and its spatial Fourier transform to be *h*(*x*) and *H*(*κ*), respectively. 

Consider that the total field vanishes on the ocean surface, i.e., the Dirichlet boundary condition. We will follow the SPM approach of Rice [9], which is to represent the coherent scattered field *E_s_* to be the combination of a perturbation series, and obtain

(1) $$\begin{array}{l} E_{i}\left(\bar{r}\right)\;=\;\exp\left(i\left(k_{i\;x}x-k_{i\;z}z\right)\right),\;where\;k_{i\;x}=\;k\sin\theta_{i},\;and\;k_{i\;z}\;=\;k\cos\theta_{i}.\\ E_{s}\left(\bar{r}\right)\;=\;E_{s}^{0}\left(\bar{r}\right)+E_{s}^{1}\left(\bar{r}\right)+E_{s}^{2}\left(\bar{r}\right)+\ldots \;=\;\int_{-\infty }^{\infty }d\kappa \exp\left(i\left(k_{s\;x}x+k_{s\;z}z\right)\right)\left[E_{s}^{0}\left(k_{s\;x}\right)\;+\;E_{s}^{1}\left(k_{s\;x}\right)\;+\;E_{s}^{2}\left(k_{s\;x}\right)\;+\;\ldots \right],\\ where\;k_{s\;x}\;=\;k_{i\;x}+\kappa ,\;and\;k_{s\;x}^{2}\;+\;k_{s\;z}^{2}=\;k^{2}. \end{array}$$

We note that *k* is the field wavenumber, *κ* is the spatial frequency of the ocean surface wave, *E_s_*^0^ would be the field from a flat surface, and *E_s_^i^* is the scattered field depending on the *i*-th power of the ocean surface elevation and its gradient. Further, we suppose that *h*(*x*) is sufficiently smooth, and thus the scattered field is composed only of outgoing plane waves at *z* higher than the maximum surface elevation *z*_0_. In practice, only the first two orders of the expansion will be calculated and used, and their spatial Fourier transforms are

(2) $$\begin{array}{l} E_{s}^{0}\left(k_{sx}\right)\;=\;-\delta \left(k_{s\;x}\;-\;k_{i\;x}\right)\;=\;-\delta \left(\kappa \right),\;and\\ E_{z}^{1}\left(k_{s\;x}\right)\;=\;i\;2\;k_{i\;z}\;H\left(k_{s\;x}\;-\;k_{i\;x}\right)\;=\;i\;2\;k_{i\;z}\;H\left(\kappa \right)\;. \end{array}$$

The SPM approach is based on formulating the scattering as a partial differential equation-boundary value problem. And, the underlying assumption of the SPM is therefore that the applied surfaces should have small surface height variation, small surface slopes, or incident angles being near the grazing angle. Leader [15] demonstrated that the SPM is actually a special case of the Kirchhoff approximation and agrees with the experimental data when the incident electromagnetic waves are near the grazing angle. This accords with most of the seaborne GPS&SBAS-R observations in this study. 

Meanwhile, ocean surface waves can exist over a range of sales from swell to capillary waves. An ocean-surface wave spectrum can be viewed as the distribution of ocean-wave energy density with respect to spatial frequency *κ*. The theoretical hypothesis of a power-law shape, i.e., *κ^−p^*, of the short-scale spatial trail is based on the physical processes of wave generation, wave–wave intersections and the dissipation involved [16]. The Pierson–Moskowitz spectrum formulation [17] and the Joint North Sea Wave Project (JONSWAP) spectrum formulation [18] have a spectral index value of −4 on spatial frequency *κ* and are quite known models to represent the spectrum of fully developed seas or the equilibrium form of ocean wave spectrum. More studies have found the spectral index value −*p* of *κ* between −2 and −6 [19,20,21]. In this study, we assume a power-law formulation *W*(*κ*) for the short-scale, i.e., high-spatial frequency, portion of ocean-surface wave spectrum, and

(3) $$W(\kappa )\;=\;W_{0}\;\kappa^{-p}\;,$$

where *W*_0_ is known as the spectral constant. 

**Figure 2:**
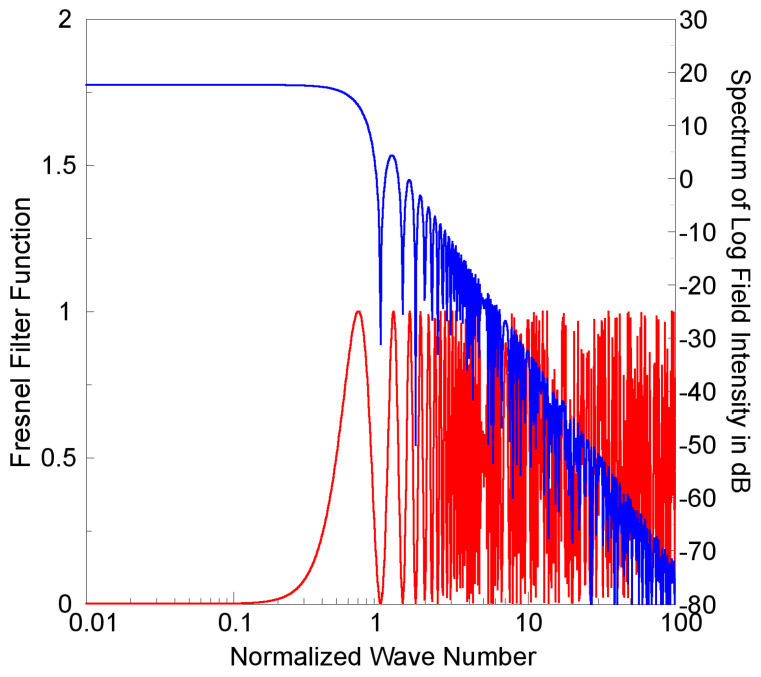
Theoretical power spectrum of GPS/GNSS-R scattered field (shown as the blue line) as a function of normalized spatial frequency *κ/κ_F_* in log scale. A Fresnel-filter function is also shown as the red line.

In the case of a plane radio wave of wavenumber *k* passing through an irregular plasma slab with spatial electron-density fluctuations, Bowhill [22] first demonstrated a two-dimensional spatial intensity spectrum, which was presented to the observer, related to the initial spectrum of diffraction signals through the irregular medium. In this study, we adapt Bowhill’s work to the case of a one-dimensional GPS/GNSS reflectometry problem. We define a distance *D* between the seaborne GPS&SBAS-R receiver and the specular point position (SPP) and obtain the spatial intensity spectrum *W_s_*(*κ*) of coherent scattered signals at the receiver as follows:

(4) $$W_{s}\left(\kappa \right)\;=\;4\;W_{0\;s}\left(\kappa \right)\;\sin^{2}\left(\frac{\lambda \left(D-z_{0}\;\csc\theta_{i}\right)}{4\;\pi }\kappa^{2}\right)\;,$$

where *W*_0*s*_(*κ*) is the spatial intensity spectrum of scattered signals at *z = z*_0_, i.e., the maximum ocean-surface elevation. Combination of Equations (2)–(4) provides a connection between the power-law spatial spectrum of ocean-surface elevation fluctuations and the power spectrum of radio-intensity scintillations observed on a seaborne GPS&SBAS-R receiver. We obtain the short-scale portion of coherent scattered signal power spectrum as

(5) $$W_{ss}\left(\kappa \right)\;=\;W_{s0}\;k_{i\;z}^{2}\sin^{2}\left(\frac{\lambda \left(D-z_{0}\;\csc\theta_{i}\right)}{4\;\pi }\kappa^{2}\right)\;\kappa^{-p},$$

where *W_s_*_0_ is defined as the short-scale spectral constant. Notably, the spatial power spectrum is controlled by two competing factors: a Fresnel-filter function, as shown as the sine square function in Equation (5), and the power-law function of ocean-surface wave fluctuation. For the case of seaborne GPS&SBAS-R observations, Figure 2 shows the Fresnel filter as a function of normalized spatial frequency *κ/κ_F_*, where *κ_F_* (*=2π/D_F_*) is the spatial frequency at the first Fresnel zone (FFZ) distance *D_F_* (=*sqrt(λD)* = ~9.75 m), corresponding to the GPS L1-band signal and a distance *D* of approximately 500 m from the R/V NOR1 to the SPP, but ignoring the maximum wave elevation *z*_0_. In Figure 2, the spatial power spectrum of simulated scattered field from Equation (5) is also shown at a spectral index *p* of 4. The short-scale spatial spectrum is generally of a power-law type with an order of *p* as decaying as *κ* increases. There is roughly a maximum around *κ_B_ (*=*κ_F/_√2*), corresponding to the first maximum of the Fresnel-filter function and also defined as the spatial break frequency. This is consistent with that of an irregularity size at the order of FFZ being most effective in causing amplitude scintillation.

## 3. System Description

**Figure 3:**
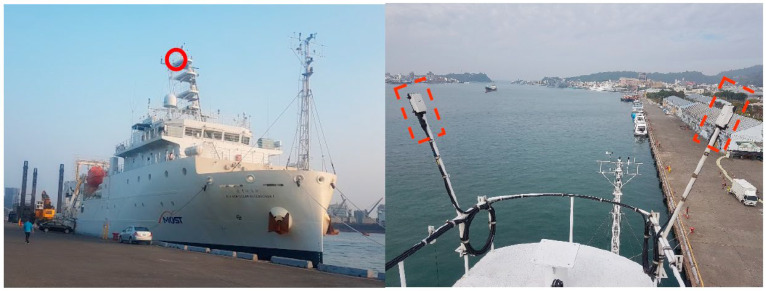
The (**left panel**) shows a picture of the R/V NOR1, where the red circle indicates the positions of two GPS&SBAS receiving antennas. The (**right panel**) shows the two GPS&SBAS-R receiving antenna installations by dotted red rectangles.

**Figure 4:**
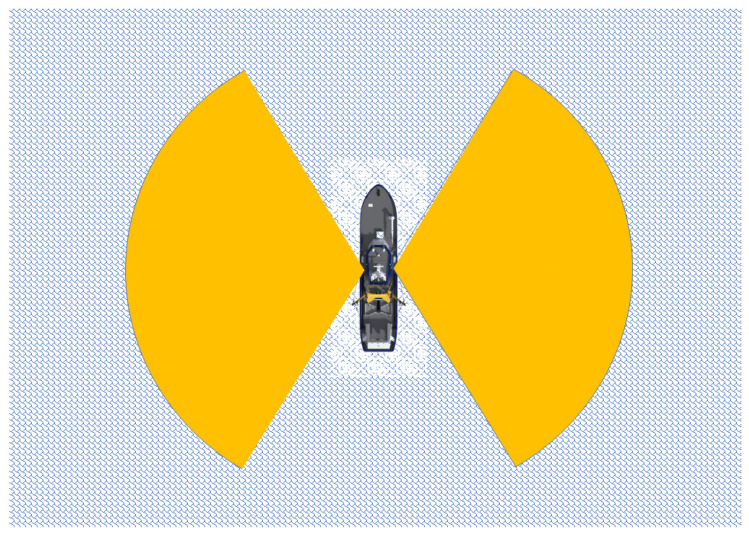
Views to the surrounding oceans of the GPS&SBAS-R observations from the R/V NOR1.

A typical software-defined GPS receiver architecture, used to track the L1-band (1.57542 GHz) Coarse Acquisition (C/A) code signals, is illustrated in Tsui [11] and also has been implemented by Tsai et al. [14]. The L1-band C/A code signals broadcast by the constellation of GPS and SBAS satellites have a known structure, consisting of a carrier modulated by a bi-phase shift keying (BPSK) pseudorandom noise (PRN) code unique to each of the GPS and SBAS satellites. We note that satellite-based augmentation systems (SBAS) have long been established to provide a positioning service for critical applications, such as commercial aviation and include the Michibiki Satellite Augmentation System (MSAS) in Japan, GPS-aided GEO-Augmented Navigation (GAGAN) in India, Wide Area Augmentation System (WAAS) for North America, and the European Geostationary Navigation Overlay Service (EGNOS). These GPS and SBAS satellites transmit right-hand circularly polarized (RHCP) radio waves, while the reflected signals from oceans are always predominantly left-hand circularly polarized (LHCP) [14,23]. In this study, a seaborne GPS and SBAS reflectometry research platform was setup on the R/V NOR1 and includes two software-defined GPS&SBAS-R systems. Figure 3 shows the pictures of the R/V NOR1 and the two GPS&SBAS-R receiving antenna installations. Two dual RHCP and LHCP GPS antenna (P/N:42G1215RL-AA-5SSF-1) produced by Antcom Corporation (http://www.antcom.com/ (accessed on 29 June 2023)) were installed, but only the LHCP connectivity and its outputs were used on each GPS&SBAS-R system. The two receiving antennas were mounted on the top of R/V NOR1 with the same altitude of approximately 25 m, but are looking down the sides in the right- and left-hand directions, separately. Thus, we call the two GPS&SBAS-R observation systems the NOR1-R and NOR1-L systems, individually. The horizontal viewing, i.e., azimuth angles from both the NOR1-R and NOR1-L systems over the ocean surface, are limited to approximately 120° shown by the yellow areas in Figure 4. The observed ocean area ranges from the nearby ocean to the SPPs with a minimum depression angle of 1°, i.e., a zenith angle of 91°, as seen from the receiving antenna. Therefore, the observed ocean area has a maximum horizontal distance of approximately 1.5 km (≈0.25/tan(1°) km) from the R/V NOR1.

Using the Antcom GPS antenna, the coherent scattered L1-band GPS and/or SBAS satellite signals can be received and then down converted, filtered, and digitally sampled by a Universal Software Radio Peripheral B200 (USRP-B200) device [24]. It is noted that, for each individual NOR1-R or NOR1-L system, we also connected a broadband radio frequency (RF) bias tee module between a USRP-B200 device and an Antcom GPS antenna. The Bias Tee module [25] is used to supply DC voltages to bias RF circuits. In this study, a sampling rate of 4 MHz was used in digitizing the received signals. This corresponds to be higher than the Nyquist frequency, i.e., 2.046 MHz, of GPS and SBAS C/A-code chip rates. After that, the sampled digital and complex signals sent to the PC host physically include different baseband PRN C/A codes from all reflectively visible GPS and SBAS satellites. These reflectively visible satellites are defined as the GPS and SBAS satellites that have specular reflections located in the targeted ocean surface regions, as shown in the yellow areas of Figure 4 for the R/V NOR1. We note that, for each seaborne GPS&SBAS-R observation, the corresponding SPP can be estimated by the satellite two-line element (TLE) data and the known R/V NOR1 position. 

Furthermore, the basic GPS and SBAS C/A code signal acquisition functions in the software algorithms are implemented by applying Fourier transform to circular correlation calculation [11,14]. Such signal acquisition could determine the received C/A code signal amplitudes, its starting point (or chip delay) compared with the local replica of PRN C/A code, and the associated satellite Doppler and receiver frequency offset of each reflectively visible GPS and/or SBAS satellite. Subsequently, the estimated time delay and associated satellite Doppler and receiver frequency offset are used to track the coherent scattered signals from a targeted satellite. The acquired and tracked circular correlation values were recorded as time-series field amplitude data from one GPS&SBAS-R observation for further data processing. The detail block diagram of a software-defined GPS-R receiver can be found in Figure 3 of [14], and the corresponding signal-acquisition algorithm was also described and explained there. Moreover, signal tracking starts performing demodulation in order to extract the satellite navigation messages which are not discussed in this study. We note that, compared with usual commercial GPS receivers, a software-defined GPS receiver could offer added flexibility and versatility by implementing most functions in software. Another advantage of a software-defined GPS receiver is a maximum signal acquisition rate of 1000 Hz due to the L1-band C/A code duration at 1 millisecond and is much higher than that of a typical commercial GPS receiver. It is important that a high signal-acquisition rate can provide our GPS&SBAS-R system better capability to detect short-scale, ocean-surface waves.

## 4. Experimental Results and Validations

**Figure 5:**
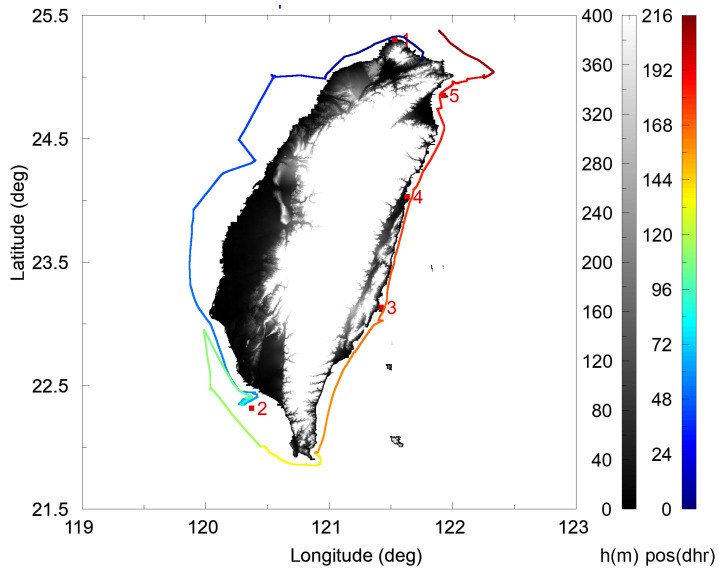
The shipping route of the R/V NOR1 during the GPS&SBAS-R experiment dated from DOYs 349 to 357, 2021, and colored in hours since the start. The red squares represent the validation locations of five CWB buoys from No. 1 to 5, and the digital terrain map of Taiwan is also shown.

In this study, a seaborne GPS&SBAS-R experiment on board the R/V NOR1 was performed over nine days from 15 to 23 December, i.e., day of year (DOY) 349 to 357, in 2021, and both the NOR1-R and NOR1-L systems simultaneously executed one 65 s duration observation every two minutes. Figure 5 shows the tracks of the R/V NOR1 positions during this experiment and also the validation locations of five Central Weather Bureau (CWB) buoys, which have a nearest distance less than 10 km from the R/V NOR1 track, separately. The five CWB buoys are at Fugui Cape Station (FCS; #1: 25.304° N, 121.534° E), Xiao Liuqiu Station (XLS; #2: 22.316° N, 120.372° E), Chenggong Station (CS; #3: 23.132° N, 121.420° E), Hualien Station (HS; #4: 24.031° N, 121.633° E), and Guishan Island Station (GIS; #5: 24.849° N, 121.926° E). We note that scintillation measurements are recorded most often as time series. The temporal variation is usually generated by a combination of transmitter motion, receiver motion, ocean-surface wave drift, and ocean-surface structure evolution. In this study, the ocean-surface structure evolution factor has been neglected because the structures of primary interests are effectively stationary over a short recording time of sixty-five seconds. The effects of transmitter and receiver motions can be included by the consideration of SPP motion and estimated by the satellite TLE and the R/V NOR1 positioning data. As described in Equation (5), the spatial power spectrum of the scattering field pattern could be measured with a suitable spaced-antenna array. In practice however, the motions of targeted ocean surface and SPP cause the spatial scattering pattern to be observed conveniently by a point receiver, as the NOR1-R or NOR1-L system used in this study.

It is now considered that, as shown in the simulated theoretical power spectrum of GPS/GNSS-R scattered field as Figure 2, an intensity scintillation spectrum of coherent scattered signals can be characterized by a spatial break frequency *κ_B_* (~0.7 *κ_F_*) and a fitted power-law function with spectral index *p* to the short-scale portion of the derived spatial power spectrum. The temporal variations of coherent scattered GPS and SBAS signals received by a seaborne receiver can be considered as the result of radio beam scanning along the SPP tracks over the ocean. Therefore, the signal acquisition frequency *f_s_* on GPS&SBAS-R observations can be transferred to the spatial signal-acquisition frequency as $\kappa_{s}\;=\;{2\pi \;f_{s}/v}_{r}$, where *v_r_* is the relative ocean-surface wave speed with respect to the moving SPP, as defined as follows.

(6) $$v_{r}\;=\;v_{w}\;-\;\hat{k}•{\bar{v}}_{SPP}\;,$$

where *v_w_* is the ocean-surface wave speed, ${\bar{v}}_{SPP}$ is the SPP velocity, and $\hat{k}$ is the normal ocean-surface wave vector that could be obtained from the CWB buoy database. Therefore, we expect a temporal breaking frequency of the intensity scintillation spectrum to be as follows

(7) $$f_{B}\;=\;\frac{v_{r}}{\sqrt{2}\;D_{F}}\;=\;{f_{B}}^{\prime }\;-\;\frac{\hat{k}•{\bar{v}}_{SPP}}{\sqrt{2}\;D_{F}}\;,$$

where *f_B_′* is the effective breaking frequency defined as $v_{w}/\sqrt{2}\;D_{F}$. In this study, the use the high-sampling, software-defined GPS&SBAS receiver, which provides reflectometry measurements with a signal acquisition rate at least twenty-fold higher than the corresponding Fresnel frequency, which can be estimated by $f_{F}\;=\;v_{r}/D_{F}$ and could approach 5 Hz at the critical case of a relative ocean-surface wave speed at 30 m/s and at a 60 m SPP-to-receiver distance. This makes sure the maximum spatial frequency on GPS&SBAS-R signal acquisition to be also at least one order larger than the expected spatial beak frequency of the coherent scattered-signal spectrum. 

**Figure 6:**
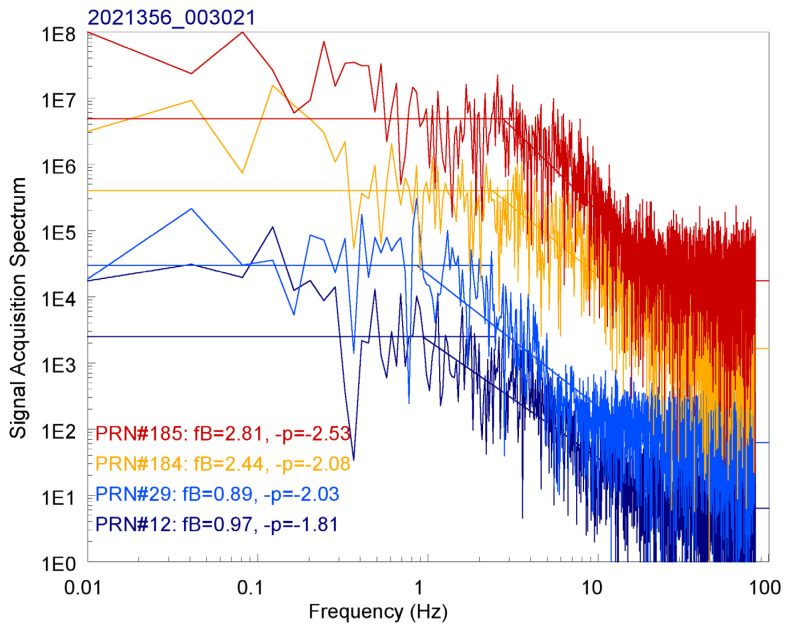
An example log–log plot on the relative Fourier power spectra with respect to four time series of GPS&SBAS-R signal acquisition data recorded by the NOR1-L system on DOY 356, 2021. The derived break frequencies and spectral indexes of the signal spectrum analyses of reflectometry observations on different GPS and SBAS satellites are also shown.

As described, we applied the circular correlation technique to C/A code signal acquisitions to determine the coherent scattered field amplitudes from all reflectively visible GPS and SBAS satellites of each reflectometry observation. Usually, two to five time-series (65 s) sets of coherent scattered field amplitudes from different navigation satellites can be obtained during one GPS&SBAS-R observation by the NOR1-R or NOR1-L system. Each time, the series of field amplitudes consequently exhibit complex scattered signals from irregular undulations on the targeted ocean surface. We propose to use spectral analysis on each obtained time series of coherent scattered field amplitudes and derive the corresponding break frequency and spectral index of each power spectrum to represent the statistical properties of targeted ocean surface. Figure 6 shows the resulting relative Fourier power spectra with respect to four time series of coherent scattered signal amplitudes, from an example GPS&SBAS-R observation recorded by the NOR1-L system. In this study, we executed L1-band C/A code signal acquisition by circular correlation calculation on the sampled complex data in every two milliseconds, i.e., at a signal acquisition rate of 500 Hz. The spectrum plot in Figure 6 focuses on the low-frequency (≤80 Hz) parts and does not show the 80–250 Hz spectra, which are similar to the spectra near 80 Hz and exhibit white noise. Notably, in this study, we have no attempts to derive the peak or mean ocean-surface wave frequency, whereas the corresponding studies can be referred to our earlier investigation [14]. As shown in Figure 6, above 30 Hz, the power spectral densities (PSD) are near the noise level. Below 2 Hz, the PSDs are a more or less flat portion at low frequencies and decay from a cut-off at a break frequency *f_B_* toward the noise level in, approximately, a linear fashion on the log–log scale shown. It indicates and confirms a power-law variation *f*^−^*^p^* of short-scale ocean-surface wave PSD as discussed. We note that the derived break frequency *f_B_* values are 1.06, 1.38, 2.99, and 2.5 Hz, and the derived spectral index *p* values are 1.69, 2.22, 1.99, and 2.26 for the coherent scattered L1-band signal spectra from the GPS and SBAS satellites, with PRN #12, #29, #184, and #185 codes, respectively. 

**Figure 7:**
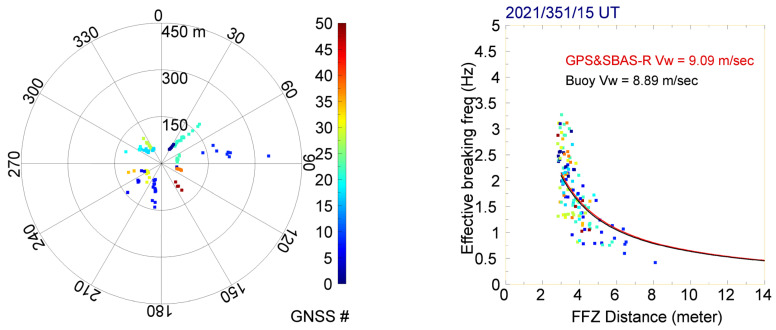
The (**left panel**) shows the azimuth-distance tracks of GPS&SBAS-R SPPs obtained from both the NOR1-L and NOR1-R system observations during 15 ± 0.5 UT on DOY 351, 2021. The (**right panel**) shows a scatterplot of the corresponding effective breaking frequencies versus the FFZ distances of all GPS&SBAS-R observations, and it also shows two rectangular hyperbola curves corresponding to a least-squares, fitting surface-wave speed and the validation surface-wave speed measured by the buoy at Xiao Liuqiu Station (XLS). The coded colors represent the different GPS&SBAS-R observations from GNSS satellite numbers, where No. 1 to 32 represent GPS satellites, and 33 to 50 represent SBAS satellites.

To evaluate the accuracy of the GPS&SBAS-R measurements, we compare the derived ocean-surface wave speeds with the buoy measurements provided by the CWB of Taiwan (https://ocean.cwb.gov.tw/V2/data_interface/datasets# (accessed on 1 June 2022)). All CWB buoys are moored buoys and provide in situ measurements of ocean-surface wave heights, wave directions, and maximum and mean wave periods at a time resolution of one hour. The validation ocean-surface wave speeds are estimated by the hydrodynamical laws [26] using the mean wave-period data and under an assumption of a deep ocean. We note that, as shown in Figure 5, the five validation buoys listed in the preceding paragraph were chosen because of having a nearest distance less than 10 km from the experimental R/V NOR1 track separately. Figure 7 shows the GPS&SBAS-R SPP tracks obtained from all the NOR1-L and NOR1-R system observations, which are located nearly 10 km away from the buoy at Xiao Liuqiu Station (XLS), during 15 ± 0.5 UT on DOY 351, 2021. The grazing incident or depression angles of GPS&SBAS observations from the NOR1-L or NOR1-R system antenna to an SPP were limited from 1° to 30° to make sure the obtained signal acquisition data had enough significant power and to also keep away from the nearby bow waves. Thus, the GPS&SBAS-R SPP could distance up to 1 km from the R/V NOR1, and the corresponding FFZ distance *D_F_* is approximately from 3.5 to 13 m. As expected, the smaller the observed depression angle is, the longer the SPP distance between two continuous GPS&SBAS-R observations. Therefore, most of the SPPs are located nearby the R/V NOR1 track. Because of the distribution of SPP during one validation is not wide and within a few tens kilometers, we assume the targeted ocean-surface wave speeds are the same. 

**Figure 8:**
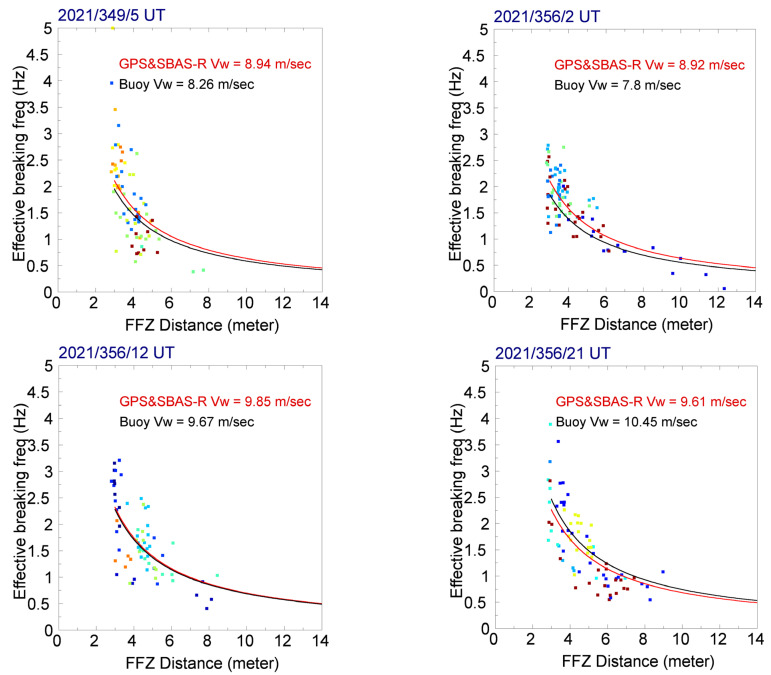
As in the (**right panel**) of Figure 7 but the results of respective GPS&SBAS-R observations nearby Fugui Cape Station (in the **upper-left panel**), Chenggong Station (in the **upper-right panel**), Hualien Station (in the **lower-left panel**), and Guishan Island Station (in the **lower-right panel**).

The right panel of Figure 7 shows a scatter plot between the FFZ distances and the derived effective breaking frequencies using Equation (7). We note that the scatter plot approximately conforms to a rectangular hyperbola equation, $v_{w}\;=\;\sqrt{2}\;{f_{B}}^{\prime }\;\times \;D_{F}$. The figure also shows two rectangular hyperbola curves corresponding to a least-squares, fitting surface-wave speed from GPS&SBAS-R observations and the validation surface-wave speed measured by the buoy at Xiao Liuqiu Station (XLS). As shown in Figure 8, four more scatter plots between the FFZ distances and the derived effective breaking frequencies are compared with validation surface-wave speeds measured by Fugui Cape Station (FCS), Chenggong Station (CS), Hualien Station (HS), and Guishan Island Station (GIS). The specific comparison results from the five CWB buoy stations, referring to the GPS&SBAS-R observations, obtained during the R/V NOR1 experiment in 2021, are summarized in Table 1. They include the recorded ocean surface-wave speeds from the five buoy stations and the mean differences and standard deviations compared with the corresponding experimental GPS&SBAS-R observations obtained on the R/V NOR1. It can be observed that the obtained surface-wave speed differences are more dependent on the experimental R/V NOR1 speeds in contrast with the surface-wave speeds or the distances between each buoy station and the experimental R/V NOR1. It is reasonable that the higher NOR1 speed would decrease the ship movement stableness and deviate the receiving antenna locations during a coherent GPS&SBAS-R observation period. This will increase the errors in the calculations of FFZ values and also the derived surface-wave speeds.

## 5. Discussions

In this study, the research platform has been built with two software-defined GPS&SBAS-R receivers looking down the side of the surrounding oceans in the right-hand and left-hand viewing directions of the R/V NOR-1. Each receiver can acquire and track L1-band C/A code signals from GPS and SBAS satellites and obtain time-series complex amplitude data. The seaborne GPS/GNSS-R techniques have been limited by measurements of coherent scattered GPS/GNSS signals because of small signal footprint areas over the ocean. It induces failings of time-delay waveform or DDM data product. Therefore, we propose the radio scintillation theory to be applied on such coherent scattered signal spectrum analysis, and we believe that this is the first use of radio scintillation theory on GPS/GNSS-R observations. Referring to the signal intensity spectrum results, as shown in Figure 6, these deployments were designed to understand the motions of ocean surface waves scaling from 0.2 to 200 m in wave length. We note that the physical characteristics of meter-scale and less-meter-scale ocean-surface waves cannot be measured by such traditional devices as buoys or capacitance gauges. Meanwhile, we also obtain a rectangular hyperbola relationship between the FFZ distances of seaborne GPS&SBAS-R observations and the effective breaking frequencies of corresponding signal intensity spectra with respect to ocean-surface wave speed. The FFZ distances from GPS&SBAS-R SPP are distributed from 3.5 to 13 m in this study, and the expected effective breaking frequencies should be approximately from 1.6 to 6 Hz at the critical case of a relative ocean-surface wave speed 30 m/s. When the receiver altitude is higher, the expected breaking frequency is lower. For the applications of space-borne GPS/GNSS-R observations at 400 km altitude, the expected effective breaking frequencies could be less than 0.15 Hz and cannot be retrieved at the critical case of a relative ocean-surface wave speed 30 m/s. We conclude that such radio scintillation analysis technique cannot be applied to ocean-surface wave measurements based on space-borne GPS/GNSS-R observations. 

Furthermore, the motions of a ship are usually defined by six displacements [27] including surge, sway, and heave terms in translational motions, and roll, pitch, and yaw terms in rotational motions. It means that the posture and gesture of a moving ship are complicated, and thus the FFZ distance of a GPS&SBAS-R SPP is difficult to be precisely estimated. It induces that the derived ocean-surface wave speed by a rectangular hyperbola fitting has reasonable error. This conforms to the comparison and validation results with the CWB buoy measurements, as shown in Figure 7 and Figure 8 and Table 1. We expect further ship displacement analyses to improve FFZ distance estimation in future studies.

## 6. Conclusions

In this study, the roles played by coherently scattering and irregular ocean surfaces through the Fresnel filtering effect on radio intensity scintillation observations have been firstly delineated. We have analyzed and simulated the coherent scattered signal spectrum with comprehensive theories in order to facilitate the interpretation of time-series GPS&SBAS-R signal measurements and their retrievals in terms of ocean state parameters, such as ocean-surface wave speed and spectral index. Software-defined GPS&SBAS receiving systems are used to provide high-signal-acquisition rate data and obtain a reliable high-frequency part of the signal intensity spectrum. A general power-law model on the short-scale spatial trail of obtained ocean wave spectrums is appealing for the statistical characterization of intensity scintillation that agrees well with seaborne GPS&SBAS-R observations. The power-law-form spectral characterization of turbulence leads a compact parameterization and is characterized by a break frequency parameter and a spectral index. Derived effective breaking frequencies and FFZ distances are also conformed to a rectangular hyperbola equation with respect to ocean-surface wave speed. However, advanced ship displacement analysis is necessary to improve GPS/GNSS-R FFZ distance estimation and further ocean-surface wave speed determination in future studies.

## Figures and Tables

**Table 1:** The specific GPS&SBAS-R observation comparison results from the five CWB buoy stations.

Buoy Station (#)	Station Coordinates	Ob. Time (UT/DOY in 2021)	Wave Speed (WS, m/s)	Mean Distance to NOR1 (km)	Mean WS Difference of GPS&SBAS-R (m/s)	WS Deviation of GPS&SBAS-R (m/s)	Mean NOR1 Speed (m/s)
FCS (#1)	25.304° N, 121.534° E	5/349	8.26	5.06	0.68	3.25	4.62
XLS (#2)	22.316° N, 120.372° E	15/351	8.89	10.16	0.2	2.36	0.70
CS (#3)	23.132° N, 121.420° E	2/356	7.8	5.51	1.12	2.37	4.75
HS (#4)	24.031° N, 121.633° E	12/356	9.67	8.33	0.18	2.77	1.16
GIS (#5)	24.849° N, 121.926° E	21/356	10.45	10.45	−0.83	2.85	4.15

## Data Availability

The raw 4.096-MHz I-Q data and corresponding time-series signal acquisition results obtained on DOY 356, 2021, from the NOR1-L and NOR1-R systems are publicly available at http://140.115.111.212/G%3A/ (accessed on 30 December 2021).
